# When Fathers Begin to Falter: A Comprehensive Review on Paternal Perinatal Depression

**DOI:** 10.3390/ijerph17041139

**Published:** 2020-02-11

**Authors:** Antonio Bruno, Laura Celebre, Carmela Mento, Amelia Rizzo, Maria Catena Silvestri, Rosa De Stefano, Rocco Antonio Zoccali, Maria Rosaria Anna Muscatello

**Affiliations:** 1 Department of Biomedical and Dental Sciences and Morphofunctional Imaging, University of Messina, 98125 Messina, Italy; lallacelebre@gmail.com (L.C.); cmento@unime.it (C.M.); zoccali@unime.it (R.A.Z.); mmuscatello@unime.it (M.R.A.M.); 2 Psychiatry Unit, Polyclinic Hospital University of Messina, 98125 Messina, Italy; amrizzo@unime.it (A.R.); mariacatenasilvestri89@gmail.com (M.C.S.); rsdestefano@libero.it (R.D.S.)

**Keywords:** paternal perinatal depression, fatherhood, mood disorders, depression

## Abstract

The transition to parenthood is considered to be a major life transition that can increase the vulnerability to parental depressive disorders, including paternal perinatal depression (PPND). Although it is known that many fathers experience anxiety and depression during the perinatal period, PPND is a recent diagnostic entity and there are not enough published studies on it. Accordingly, its prevalence and epidemiology are still not well defined, although the majority of studies agree that PPND is less frequent than maternal perinatal depression and postpartum depression. Nevertheless, PPND is different from maternal perinatal mental health disorders, usually, fathers have less severe symptoms, and mood alterations are often in comorbidity with other affective disorders. Despite the absence of DSM-5 diagnostic criteria and the fluctuation of prevalence rates, clinical symptoms have been defined. The main symptoms are mood alterations and anxiety, followed by behavioral disturbances and concerns about the progress of pregnancy and the child’s health. Moreover, PPND negatively impacts on family functioning, on couples’ relationships, and on family members’ well-being. The aim of this paper is to present an overview of the current understandings on PPND and the potential screening, prevention, and treatment options.

## 1. Introduction

Pregnancy and early parenting are major life transitions that increase the vulnerability to psychological distress and the onset or relapse of psychiatric disorders, mainly depression, for both parents [1,2]. The issue has been thoroughly studied in the literature, with an almost exclusive focus on the mental health of women and the need for identification of depression during pregnancy and after the birth [3]. Postpartum depression (PPD), with a global prevalence of approximately 17% [4], is recognized as a subtype of a major depressive disorder (MDD) in the Diagnostic and Statistical Manual for Mental Disorders, Fourth Edition (DSM-IV) [5], which assigned a “with postpartum onset” specifier to episodes of depression that occur within four weeks of delivering a child. An important update on the topic is that in DSM-5 [6] the specifier has changed for “with peripartum onset”, thus encompassing depressive episodes occurring during pregnancy, as well as in the four weeks following delivery. This extension of the time period acknowledges the evidence that rates of maternal depression are higher during pregnancy than during the postpartum period, and that the majority of cases of PPD are preceded by depression during pregnancy, as shown by longitudinal studies [7]. Moreover, most experts in the field have argued that the time of onset for the relevant symptoms in the postpartum period should be extended from six months to one year after delivery [8].

Due to its substantial impact on the well-being of both mothers and newborns, with children exposed to maternal depression being more at risk for impaired emotional and cognitive development as well as depressive disorders in adolescence and adulthood, maternal perinatal depression (MPND) is still receiving sustained attention by clinicians and researchers [9], and the focus of care in the majority of maternity services is on the mother-infant dyad. The mental health of fathers in the pre- and postnatal periods has traditionally received less public and clinical attention. Nevertheless, a growing body of evidence suggests that men also experience depression after the birth of a child or in the perinatal period.

Paternal perinatal depression (PPND) is not widely acknowledged or well researched, and it is not recognized as an official psychiatric disorder; according to the DSM-5, there are no official criteria to make a diagnosis of PPND [6], and there is still a lack of agreement on its defining factors, probably reflecting the composite nature of PPND and methodological problems in assessment instruments. The onset of a depressive symptomatology in fathers can occur from the beginning of pregnancy; depressive symptoms decrease shortly after childbirth but increase over the course of the first year [10]. Considering clinical features, PPND differs from maternal perinatal depression (MPND) and maternal postpartum depression (PPD), since men and women express depression and cope with it in different ways [11]. For both men and women, depression presents as a dysphoric mood with reduced activity; however, in men, exhaustion, fatigue, self-criticism, irritability, restlessness, and anger attacks prevail over low mood. Depressive symptoms are often comorbid with anxiety and obsessive disorders, and a range of somatic symptoms and complaints, along with alcohol and drug abuse, which can mask the main symptoms of PPND, are also frequent [12]. Depressed men are also more likely to display hyperactive or avoidance behavior, interpersonal conflicts, and lower impulse control than depressed women [11].

PPND prevalence is still not well defined, as documented by prevalence rates ranging from 4% to 25% in first-time fathers, up to 50% in the cases of concomitant depression in the partner [13]. This considerable variability could be due to a number of factors, such as the heterogeneity of assessment time points, gender differences in symptom presentation, location of the epidemiological studies, populations assessed, the use of screening instruments that are developed for mothers, and gender differences in accessing health-care services [14]. A meta-analysis of 43 studies showed that 10.4% of fathers and 13% of mothers experienced depression with relatively higher rates, up to 25.6%, for fathers in the three to six month postpartum period as compared with 4.8% of the general male population during a given year [15]. In this study, paternal depression was found to be moderately correlated with maternal depression, thus, confirming previous results [16]. More recently, a meta-analysis of 74 studies with 41,480 participants reported a prevalence rate of approximately 8% for paternal depression experienced during pregnancy and up to one year postpartum [17]. Results also highlighted that rates of paternal depression were conditional on rates of maternal depression, and varied based on the location of the study, with USA studies reporting prevalence estimates up to 13%. Additional variation depended on the measures used for assessing depressive symptoms, such as interviews versus self-report scales, since structured and semi-structured interviews produced a lower point estimate of depression than self-report instruments. Differently from previous studies on risk factors or correlates of perinatal depression in fathers [18], in this meta-analysis several sociodemographic and clinical variables, such as paternal age, parity, education level, and previous history of depression did not significantly moderate rates of depression, although paternal tertiary education trended towards significant moderation. 

The nature and the direction of the relationship between PPND and MPND have not yet been completely clarified; what is known is that the two conditions are significantly related, since it has been reported that maternal depression is a predictor of paternal depression [15,17], but also that early paternal depression incrementally increases the risk for continued or worsened maternal depressive symptoms through six months postpartum [8]. Hence, rather than considering parents individually, it is important to recognize the existence of an interaction between the psychological states of both members of the couple in order to provide successful interventions [19]. Increasing evidence points at the role that paternal depression has in child development and later psychosocial outcomes; similar to the maternal form, PPND can lead to developmental problems and mood or anxiety disorders in the offspring [20,21]. The effect of PPND on early childhood has been evaluated by researches with longitudinal data from around the world showing emotional and behavioral problems [22], lower social and psychological well-being [23], and internalizing behavior problems [24] in children aged three to 12 years. Furthermore, it has been shown that the risk for offspring mood or anxiety disorders did not differ according to whether the affected parent was the mother or the father, and that children where at significantly greater risk when both parents were depressed [25]. Thus, when considering the consequences that paternal depressive symptoms have on both fathers’ and mothers’ well-being, as well as on the development children, the accurate and early assessment of PPND is mandatory, although it must be taken into account that, due to the potential stigma related to paternal depression, there is a tendency to postpone the search for help in the affected fathers [20]. The traditional gender-focused approach should be supplemented by a family-focused approach, in order to ensure more effective intervention strategies for the benefits of the family unit as a whole [20].

The present narrative review aims to collect and analyze relevant literature on paternal depression in the perinatal period in order to present a concise overview of clinical features and main hallmarks of this disorder, with particular attention to risk factors and therapeutic and preventive strategies.

## 2. Methods

### 2.1. Research Strategy

PubMed and Scopus database were searched up to September 2019, using the following terms: “father” OR “paternal” AND “depression” OR “perinatal depression”. Articles were selected by title and abstract; the entire article was read if the title and abstract was related to the specific issue of PPND, and if the article potentially met the inclusion criteria. References of the selected articles were also examined in order to identify additional studies meeting the inclusion criteria.

### 2.2. Study Selection

Articles were included in the review according to the following inclusion criteria: English or Italian language, publication in peer reviewed journals or books, and quantitative and qualitative information on PPND. Articles were excluded by title, abstract, or full text for maternal depression, and for irrelevance to the topic in question. Further exclusion criteria were articles not written in English or Italian language, unpublished dissertations and theses, and case reports and series and other non-peer-reviewed material.

### 2.3. Data Extraction

**Figure 1:**
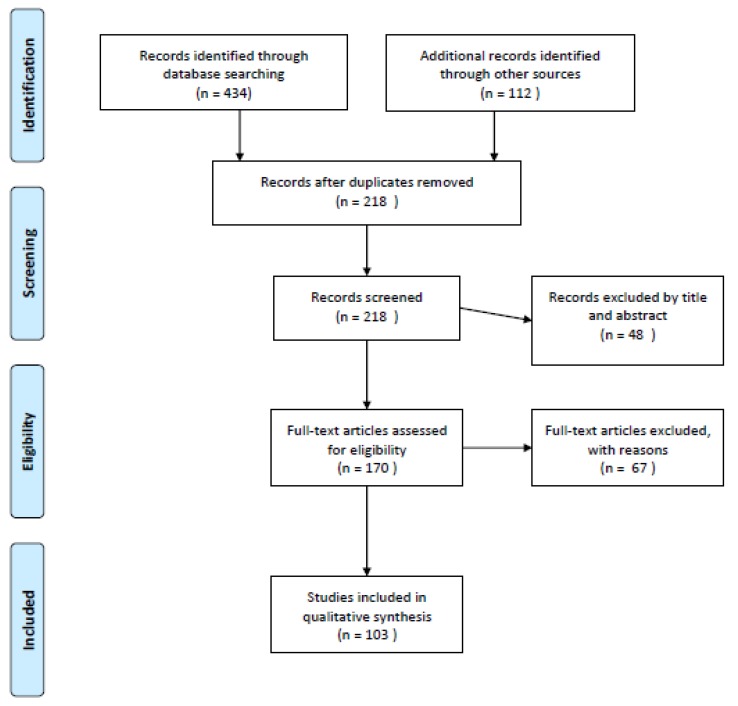
Flow diagram of the literature selection process.

Figure 1 summarizes the flowchart of articles selected for the review. The search of the database provided a total of 434 citations; 112 additional studies meeting inclusion criteria were identified by checking the reference list of the selected papers. After adjusting for duplicates, 218 records were screened. Of these, 48 studies were excluded by title and abstract, and 67 studies were excluded according to the inclusion and exclusion criteria. After the screening, a total of 103 studies assessing PPND met the inclusion criteria and were included in the review; in particular, 26 studies focused on prevalence, clinical features, and differential diagnosis, 4 studies focused on outcome, 24 studies focused on risk factors, 8 studies focused on the relationship between PPND and maternal depression, 20 studies focused on PPND influence on a child’s development, 12 studies focused on assessment and screening and 9 studies focused on prevention strategies and treatment.

## 3. Clinical Features and Differential Diagnosis

Clinical features of PPND are heterogeneous, showing less defined symptoms as compared with maternal perinatal disorders [26]. Moreover, the course of the disease is rather long, more than half of fathers with PPND during pregnancy continue to be symptomatic after six months postpartum [27]. Mood alterations and anxiety are constantly present but often underestimated. Affective symptoms include stress, hopelessness, depressive mood, irritability, restlessness, pervasive concern about the progress of pregnancy and the child’s health, loss of interests, lack of concentration, social withdrawal, sleep alterations, and decreased or increased appetite [28]. Worries and thoughts experienced by the patients can reach an obsessive intensity, with anxious and intrusive thoughts mainly directed toward the child’s health [29]. Almost 80% of fathers report recurrent thoughts about the infant’s health, development, physical safety and vulnerability, and the possibility of harming the infant through acts of negligence or deliberately [29]. Usually, intrusive thoughts and other symptoms are not spontaneously revealed by the fathers, thus, symptoms always need to be asked directly. Mood alterations are often associated with behavioral disturbances and lack of impulse control [30]. The most frequent behavioral abnormalities are the following: (a) rage attacks and aggressive behaviors toward the infant and the partner; (b) avoidance and loss of interest in family life, with increased engagement in work and/or leisure activities; (c) extramarital affairs; (d) compulsive exercise or sexual activities; (e) excessive use of videogames and internet (e.g., compulsive chatting, pornography, and sex site usage, internet addiction disorder) or gambling (via slot machine or computer) [30]. Depressed fathers are more vulnerable to heavy smoking, alcohol abuse, drug addiction, and food behavior disorders, and in some cases these unhealthy habits mask the affective disorder [31].

Furthermore, in perinatal depressive disorders, illness behavior, defined as the mode of experiencing, evaluating, and responding to one’s own health status, is often abnormal, with specific difficulties related to emotional processing and regulation [32]. Some of the affected fathers pay too much attention to physical appearance and develop hypochondria or somatic symptoms such as headaches, back pain, tachycardia, and respiratory, digestive or urinary problems [33]. Moreover, the birth of a child is a time of intense changes which often affect the couple’s sexual life [34]; rates of sexual dysfunction during pregnancy are high among both men and women [15]. Sexual problems and dysfunctions during pregnancy are often caused by the father’s anxiety of injuring the fetus [35]. Despite the fears and limits related to sexual activity during pregnancy, maintaining sexual interactions promotes well-being and intimacy between the partners [36]. Changes in the couple’s sexuality in the perinatal period are influenced by relational, psychological, and biomedical factors. Among psychological factors, the quality of prenatal sexual activity, mental disorders, especially depressed mood, and desire or excitement disorders play a significant role in affecting sexual life in the peri- and the postnatal period [36]. Genital pain and dyspareunia are biomedical factors associated with impaired sexuality; however, both these factors can also depend on affective symptoms (anxiety or depression) related to the pregnancy or to the postpartum period [35]. On the one hand, depression is an important predictor of the reduction of sexual desire and satisfaction since it is associated with apathy, low self-esteem, specific concerns about body image, fatigue, and other symptoms that greatly affect sexual interest [36]. On the other hand, poor sexual performance can also lead to depression and cause relationship conflicts. Several psychiatric conditions can occur in comorbidity with PPND. Concerning differential diagnosis, schizophrenia, bipolar, and other disorders must always be considered and eventually excluded [37]. Furthermore, PPND must be distinguished from the “Couvade Syndrome”, a disorder characterized by somatic symptoms, such as nausea, swelling, tension, and abdominal pain and by the activation of regressive or passive feminine behaviors with peculiar concerns in pregnancy [38].

## 4. Outcome

Research shows that perinatal mental illness confers long-term psychiatric and medical vulnerabilities for the mother, the offspring, and the family [39]. In women, risk of recurrence of ante- and postnatal psychiatric disorders outside of the perinatal period is considerable, since a significant percentage of mothers who experience perinatal affective disorders develop recurrent long-term mood disorders, both unipolar and bipolar depression; furthermore, postpartum depression is one of the greatest causes of maternal mortality and long-term morbidity [39]. Along with the longitudinal course, trajectories of perinatal disorders in women have also been studied, whereas fathers’ depression trajectories have received much less attention from research. A longitudinal study aimed at analyzing trajectories of depressive symptoms from the third trimester of pregnancy to one year after childbirth in a sample of 126 first-time fathers identified three distinct symptom trajectories, named “resilient” (stable low depressive symptoms, 52% of the sample), “distress” (stable moderate, 37%), and “emergent depression”. This latter class represented the high-risk condition, with symptoms reaching the threshold values at one year after childbirth, and included 11% of the subjects, a percentage fitting the rates for paternal PPD found in other studies [40]. A Finnish study among normative and former infertile couples (N = 773) followed up to 12 months postpartum found five paternal depressive and psychological distress trajectories in 10% of new fathers, stable low (79%), and moderate increasing (9%) levels of symptoms, and prenatal (5%), early fatherhood (3%), and heterogeneous high levels of (4%) depressive symptoms and psychological distress [41]. To date, the longest follow-up study on PPND has evaluated 1670 mothers and 1604 fathers from the Finnish CHILD-SLEEP birth cohort, up to 24 months postpartum [42]. Three stable depressive symptom trajectories were found in fathers, low (74.9% of the male participants), moderate (22.6%), and high (2.6%). It was also shown that the percentage of fathers presenting clinically significant depressive symptoms doubled from prenatal (or three months) to two years postpartum. For both parents, strong predictors of higher depressive symptom trajectories were the presence of prenatal and previous psychopathology. As stated by the authors, stability was one of the main features of the identified trajectories, thus, in the presence of depressive symptoms, it could be hypothesized that stability of trajectories stood for chronicity.

However, based on the very few longitudinal studies actually available, many unresolved questions persist. It remains unclear whether PPND represents a risk for other mental disorders beyond two years postpartum, or whether PPND is a subsyndromic or prodromal stage of major depressive disorders or could increase the risk of future mood disorders or other clinical entities.

## 5. Correlation between Paternal and Maternal Depression and Influence on Child’s Development

During the perinatal period, the emotional states of mothers and fathers influence each other; thus, significant correlations between paternal and maternal perinatal depressive disorders are not unexpected [13,15,30,43]. Research conducted on the partners of 40 women hospitalized in a mother-baby unit for a postpartum affective disorder showed that 42% of fathers suffered from anxiety disorders, episodes of major depression, and low psychosocial functioning [44]. In a Canadian study, 50 fathers whose partners had perinatal depression were compared with a control group whose partners did not show clinically significant signs and symptoms of affective disorders [26]. In the first group, 24% of the subjects were diagnosed with a psychiatric disorder (depression, anxiety, or adjustment and somatization disorders) versus 10% in the control group. An Italian study confirmed that the partners of mothers who experienced postpartum affective disorders tended to be more anxious, depressed, and irritable; hypochondria, somatizations, and dysphoria were also found [45]. According to systemic theories, any factor affecting a family member has an effect on other family members. Therefore, living with a depressed or anxious family member can have a depressing effect due to the mutual influence of psychological states. However, the correlation between paternal and maternal depression can be explained as a consequence of the failure of the safe protective function performed by the father towards the partner [46]. Fathers with an insecure attachment, too worried, anxious, depressed, or hostile are at a disadvantage in building couple and father-child relationships [43]. The quality of paternal care is important for a child’s cognitive, emotional, and social development [2]; the impact of parents’ depressive symptoms on infant development is significant, and even more detrimental when both parents are depressed [47]. Infants and adolescents who experience psychopathological disorders, such as attention-deficit and hyperactivity disorders (ADHD), conduct disorders, oppositional defiant disorder (ODD), anxiety, and depression [22], are often children of depressed parents [48]. The early relationship of the newborn with both parents plays a central role in the child’s cognitive development, including mentalizing faculties, and in the ability to control and regulate emotions [33]. Furthermore, parental depressive disorders are associated with inadequate parental behaviors and with an altered perception of the child’s health status and needs. The Early Childhood Longitudinal Study-Birth Cohort (ECLS-B) [49] evidenced that, when both parents are depressed, there is a limitation in educational or recreational activities, i.e., playing with the child, reading or telling him stories, and singing songs. However, only paternal depression seems to have a negative impact on the richness of the child’s expressive vocabulary at 24 months of life [50]. Despite this, the partners of depressed mothers are more involved in positive interaction with the child [51]. Indeed, on the one hand, fathers with a secure attachment pattern could have an early positive influence on the child, compensating for the deficiencies in the mother–child interaction. On the other hand, insecure fathers present poor mentalizing abilities or behavioral and psychological disorders which affect the mother-child bond hindering the psychophysical development of the child [52]. Lastly, PPND seems to be an independent risk factor for child neglect and maltreatment [53].

## 6. Etiopathogenetic Hypotheses

Several etiopathogenetic hypotheses have been proposed to explain the onset of PPND. Psychoanalytic theories hypothesize a reactivation of pre-oedipal or oedipal conflicts. According to this, authors have described depression as the activation of the defenses of men to cope with the anxiety caused by the birth of a child. Others have focused on the hormonal factors and on the influence of psychosocial risk factors in promoting depressive symptoms, for example, relationship conflicts, high parental stress, and poor social support. Table 1 summarizes the main theories about the predisposing and risk factors associated with the onset of PPND.

### 6.1. Psychodynamic Hypotheses

Psychoanalysts have long considered the possibility that men, as well as women, can develop affective disorders related to pregnancy. “Depressive reactions related to parenthood” was the first study on fatherhood-related psychological disorders [54]. The study highlighted the following three common features of depressed fathers: Regressive attitudes of dependence and jealousy due to rivalry towards their own child, unconscious incestuous fantasies linked to a conflict with their own mother and symbolically transposed on the partner, and depression as a defense against anxiety and frustration induced by the birth of the infant and the subsequent reactivation of the oedipal conflict [54]. Prior investigations have suggested that pregnancy is a vulnerability factor for future fathers in terms of identification with their own fathers, substantial dependence on their partners, or the manifestation of conflicts related to latent homosexuality [55]. Other studies indicate that empathy with the child could promote access to mnemonic traces hidden by unconscious defensive mechanisms [56]. The French psychoanalysts Luca and Bydlowski (2001), proposed that, differently from maternal depression which was linked with narcissistic disturbances, PPND was related with oedipal conflicts, since fathers do not experience the bodily and narcissistic link with the child, given by pregnancy, childbirth, and breastfeeding. Following the psychodynamic perspective, there are different interpretations of the paternal depressive reaction. First of all, it can be a defense against the reactivation of memories of distressing childhood experiences. Alternative explanations involve ambivalent and conflicting relationships of men with PPND with their own fathers, envious unconscious reactions towards the generative role of the partner, expressed through competitive and devaluating attitudes, and narcissistic imbalance [57]. Narcissistic wounds and tendencies can be expressed through the fear of growing old or dying, or the fear of losing the attention of the partner, that can generate jealousy towards the child, perceived as a rival. Lack of sexual desire, low self-esteem, feelings of loss and abandonment, and the inability to live without the exclusive love of the partner are also frequent [46].

### 6.2. Psychosocial Risk Factors

The development of paternal depressive symptoms has been associated with several psychosocial risk factors, such as the onset of perinatal depression in the partner (maternal depression), the presence of couple conflicts and marital dissatisfaction, the perceived stress level, and individual features, essentially personality traits and childhood history [30,43].

#### 6.2.1. Maternal Depression

The manifestations of PPND are significantly related to the partner’s depression. Maternal depression is the strongest predictor for the development of paternal depression, anxiety, and psychological distress, with rates ranging from 24% to 50% [13]. There is accumulating evidence that partners of perinatal depressed women reported feelings of anger, helplessness, fear, confusion, along with a sense of isolation and uncertainty about the future, loss of intimacy, and disruption of family social and leisure activities [58]. Symptoms of depression, anxiety, fatigue, worry, distress, guilt, despair, and sleep disturbances in fathers have also been considered as consequences of the caregiver burden derived from the constant care and attention of the depressed partner, including reported stress and fatigue from increasing demands which additionally cause feelings of anger and resentment [59].

#### 6.2.2. Couple Conflicts and Marital Dissatisfaction

An unsatisfactory couple relationship emerged in studies conducted on both maternal [60] and paternal depression [60]. Low levels of satisfaction and marital cohesion associated with high levels of perinatal distress can occur [60]. An increase in the need for protection, with the activation of the attachment figure, is hypothesized as a trigger for anxiety and depression during the transition to parenthood, especially in couples with insecure attachment. In particular, couples composed of a fearful-avoidant mother and an avoidant-distancing father were found to be the most vulnerable to develop perinatal depression [61]. The fearful-avoidant subjects, however, tend not to seek protection, even in the case of difficulties [61]. The avoidant-distancing subjects tend to react with hostility and to escape emotionally and physically from their partner’s requests for protection [61].

#### 6.2.3. Perceived Stress Level

High levels of stress maintained throughout the pregnancy up to 18 months after childbirth are considered predictive of depressive symptomatology. In particular, a “difficult” newborn (e.g., requiring constant attention, not sleeping, crying, fed with difficulty) is perceived by parents as more stressful [62].

#### 6.2.4. Personality Traits and Childhood History

PPND seems to be influenced by several personality traits, psychological features, and childhood antecedents of both mother and father, such as the presence of depressive traits, neuroticism, low level of extraversion, immature defensive styles, low educational level, and history of physical or sexual abuse [63].

Other psychosocial risk factors are an unexpected pregnancy, previous abortions or the death of a child, frustrated expectations related to the birth (e.g., the desire of a child of a different sex), and insufficient information about childbirth and pregnancy, low self-esteem, and the perception of poor parenting skills [64]. Moreover, fathers’ sense of mastery influences the perception of family functioning [65] in such a way that being unemployed or having work-family conflicts are perceived as a failure to achieve paternal role competence and has been found to be significantly predictive of paternal mental health problems [3]. Even the young or advanced ages represent a risk factor [66,67]. Surprisingly, the father’s previous psychiatric history was not significantly correlated with perinatal affective disorders [68].

### 6.3. Biological Risk Factors

Although there is extensive literature on biological risk factors for maternal depression, there is little research on PPND. As well as maternal depression, PPND can also be influenced by hormonal changes occurring during a partner’s pregnancy. Several biological factors have been investigated to explain the onset of PPND. Low testosterone levels correlate with both paternal and maternal depressive symptoms [69,70]. Testosterone levels decrease during pregnancy and for several months after childbirth, in order to reduce aggressiveness and to promote attachment with the child; indeed, the father expresses greater sympathy and caring attitudes towards the newborn [71,72,73]. PPND could also be related to a reduction of estrogen levels. Among men, levels of estrogens increase during the last months of the partner’s pregnancy until the first postpartum period, promoting more operational and maternal behaviors. Studies on paternal behavior in animals underline the presence of a greater number of estrogen receptors in the medial preoptic area and the arcuate nucleus of the hypothalamus, both involved in paternal conduct [72,74]. Thus, male rats most involved in parenting had higher levels of estrogen [72]. These results suggest that estrogen levels are involved in the regulation of caring paternal behavior and that receptors dysregulation constitute an important risk factor for the onset of depressed mood in fathers. Moreover, PPND could be associated with lower cortisol levels. Cortisol increases in psycho-physical stress conditions, allowing the physiological stress response regulation. Although high cortisol levels are usually associated with stress, during postpartum, in mothers they appear to be related to a higher sensitivity towards the child and a less depressed mood. Therefore, lower levels of cortisol in fathers have been found to be related to difficulties in the father-newborn emotional bond and to depressed mood [75,76]. A correlation between PPND and lower levels of vasopressin was also found. Vasopressin increases in fathers after childbirth in a similar way to the oxytocin level increase in mothers, and it plays an important role in improving the parent-child bond [77,78]. On the basis of these few evidences, fathers with low levels of vasopressin are supposed to have difficulties in parental behaviors and greater vulnerability to depression. Finally, a link between PPND and changes in prolactin (PRL) levels has been found [72]. Prolactin levels in men tend to gradually increase during pregnancy until the first post-natal year. A reduction of PRL could cause fathers’ difficulties in adapting to parenthood, and therefore greater susceptibility to a negative mood [79].

## 7. Screening of Paternal Affective Disorders

Currently, there are no screening tools sufficiently accredited to evaluate perinatal affective disorders in males [80]. The psychodiagnostic evaluation is mostly performed through self-report questionnaires, although questionnaires are rarely supplemented by individual or couple clinical interviews. Recently, video recording techniques have been used to assess the father-child interaction and highlighting possible emotional and relational difficulties, with the advantage of being also used as a therapeutic tool.

### 7.1. Self-Administered Questionnaires

The most used specific questionnaires on affective disorders in the perinatal period are self-report tools that do not consider gender differences, and therefore are less predictable for the evaluation of affective symptoms in males. It is well known that, due to cultural reasons, social image, or gender roles men tend to hide their psychological difficulties. Rather than asking for help, they endure stress and discomfort through “externalizing” strategies, such as smoking, drinking alcohol, assuming drugs, gambling, and compulsively engaging in sports or sexual activities. The Edinburgh Postnatal Depression Scale (EPDS) [81,82] is one of the most widely used questionnaires for assessing postnatal depression. EPDS is a 10 item measure aimed at detecting patients at risk for perinatal depression. Each item is scored on a four-point scale (from zero to three), with a total score ranging from zero to 30. The scale investigates maternal emotions and thoughts and includes items related to depression, guilt, anhedonia, anxiety, and suicidal ideation. A cut-off score ≥10 is used to detect depressive symptoms. However, attempts to validate the EPDS in men have evidenced minor cut-off scores as compared with those used with mothers [83,84]. Nevertheless, for an early assessment of male affective disorders, assessment tools specifically tailored to gender differences and the use of externalizing strategies would warrant better sensitivity and reliability. Recently, a new screening tool for paternal affective symptoms, the Perinatal Assessment of Paternal Affectivity (PAPA), has been proposed [85]. The questionnaire investigates several symptom dimensions including anxiety, depressive symptoms, perceived stress, somatic symptoms, hypochondria, addictive disorders (smoking, drinking alcohol, taking drugs, gambling, and compulsive use of the internet), performing risky physical activities, and sleep problems [85]. Moreover, at the end of the questionnaire, the subject is asked to assess how much these aspects are related to paternity. The PAPA is a screening tool whose purpose is to identify vulnerability for affective disorders in fathers, rather than a diagnostic instrument [85]. Regardless of the administered tool, it is mandatory to integrate the psychodiagnostic assessment with information obtained from individual and couple clinical interviews in order to accurately investigate the presence and the severity of paternal affective disorder [86].

### 7.2. Audiovisual Recording Based Techniques

The direct analysis of the father-child relationship is crucial to the screening of paternal perinatal affective disorders, giving the possibility to highlight live any difficulties in the ability to take care of the child. One of the most widely used tools is the CARE-Index [87], consisting of a 3 to 5 min video recording of the spontaneous relationship between child and parents. The procedure evaluates the “parental sensitivity”, namely the adult’s ability to perceive the infant’s needs and to ensure the infant’s well-being [88]. It identifies the riskiest situations, including those in which a parent manifests an affective disorder and to evaluate the association between levels of depression and psychomotor development of the newborn [45]. Another very promising technique for the study of paternal affective disorders is the use of video recordings to evaluate the mother-father-child triadic relationship [89,90]. It analyzes the interactions that mother and father have with each other, rather than considering the mother or the father individually. Due to the use of this technique, associations between the quality of interactions of the couple and those subsequently manifested during the post-natal period have been identified in 80% of families, with continuity from pregnancy to 18 months of life [91]. These pieces of evidence led to the theory that the evaluation of the “nascent” family interactions is possible during pregnancy, and therefore this would allow early screening for possible family maladjustment, including PPND.

## 8. Prevention of Paternal Perinatal Affective Disorders

The problems in the identification and prevention of the father’s emotional problems in the perinatal period are still many, but they can be overcome. Despite the increased interest in this topic, the preventive and therapeutic aspects of male affective disorders are still neglected [30,43]. For effective prevention, diagnostic evaluation and treatment of paternal perinatal affective disorders, more specific aspects should be taken into account. First of all, the disorder should be considered within a systemic family perspective, bearing in mind that during the entire perinatal period, the mental state of the parents influences not only each other but also the development of the child. Therefore, it is almost mandatory to assess the affective state from the beginning of pregnancy and, when a parent is depressed, to carefully consider the possibility that the partner could also experience mood disorders [92]. Thus, professionals in obstetrics and gynecology, as well as pediatrics should be prepared to consider not only physical health of both parents, but also mental issues, and to be aware of the early symptoms of an affective disorder. Furthermore, they should be trained to adequately inform parents about the manifestations and characteristics of perinatal mood disorders, rather than addressing only the mother’s mental status [80,92]. Fathers, for example, are often aware of the existence of maternal perinatal depression, but they are usually uninformed about paternal mood disorders; thus, healthcare professionals should give correct information about risk factors and clinical features of depression. Likewise, it is also appropriate to inform mothers about the possibility that their partner can suffer from an affective disorder. Organizing meetings with parents and distributing informative material in consultation centers and hospitals, as well as disseminating information via the internet and the media, could be helpful. It is also crucial to recognize the importance of the father from the beginning of pregnancy, supporting him in his role, identifying his difficulties, and promoting his involvement in gynecological visits, in activities of family counseling, and assistance throughout the first year of child’s life [80,92]. Moreover, it is significant to remember that emotional distress in fathers often occurs in the form of behavioral and psychological disorders that can mask depressive symptoms. However, to better support fathers during the transition to parenthood, specific prevention programs have been developed.

A recent Canadian study on needs and opinions of men affected by perinatal depression showed that fathers prefer individual help and home support rather than group therapy [93]. One of the most interesting programs is the Paternal Perinatal Depression Initiative (PPDI) that has been developed in Australia [94]. It is a national prevention and screening program for the difficulties experienced by fathers during the transition to parenting by the use of a series of strategies. One of the most interesting is the “SMS4Dads”, which consists of contacting the fathers with free messages about practical information on the management of the most common difficulties [95]. In addition, it offers information on the typical characteristics of male affective disorders and personalized psychological assistance provided by telephonic or individual interviews with specialists. Moreover, it offers a series of internet services addressed not only to fathers, but also to family doctors, obstetrics, gynecologists, and pediatricians.

## 9. Treatment

When the patient shows significant distress or when family relationships are compromised, individual or couple psychotherapeutic intervention is mandatory, eventually associated with pharmacological treatment [13,30]. In high-risk families (e.g., abusive families, adolescent parents, drug addicts, or preterm children and children with physical illnesses) home support interventions and prevention programs involving all family members have been proposed [45]. Despite this, few controlled studies on the various psychotherapeutic models (cognitive-behavioral, psychodynamic, systemic, and psychoeducational) and on the use of antidepressant drugs treatment have been conducted [80].

### 9.1. Parent Training Techniques and Video-Feedback Interventions

Throughout the perinatal period, parent training techniques integrating cognitive-behavioral, psychodynamic, and psychoeducational interventions have been useful, including the use of video-feedback techniques. As emphasized before, the most useful interventions in the perinatal period are the audiovisual recordings of the interaction between parents and children. Watching the videos, accompanied by the therapist’s comments, suggestions, and encouragement promote parents’ sensitivity, supporting them to adopt a more appropriate attitude towards the child and the partner. Such an integrative strategy promotes the couple’s mental and relational well-being and the development of secure attachment bonds, with positive and lasting effects on the development of the child. The most widely used techniques are:

- Systematic Training in Effective and Enjoyable Parenting (STEEP), is a video-feedback protocol recording the father doing “what he likes” with his child and, then, looked at with the therapist to discuss some questions, for example, “what is the child thinking here?”
- Video-feedback Intervention to promote Positive Parenting (VIPP) is an evidence-based intervention protocol to support parenting based on the attachment paradigm and the theory of social learning. The aim is to prevent the development of behavioral problems as well as to help parents to take the child’s perspective (speaking for the child). The protocol is composed of video recordings acquired during six sessions, followed by the therapist’s proposals of various hypotheses on the reasons for the infantile behavior.
- Child Adult Relationship Experimental (CARE) Index video feedback is a video-observation tool for assessing the quality of the caregiver and child interactions [87]. The CARE-Index is able to detect adult–child interactions through the recording of a small interaction of about 3 to 5 min. The quality of attachment relationships is determined by the perception of the environmental danger and the search for self-protection. According to the dynamic maturative model (DMM) [96] attachment patterns are self-protection strategies learned from the protective figure itself, which in most cases consists of one of the parents. Although the adult protective figure is generally the mother, the CARE-Index can be used to evaluate interactions even with fathers or with all those adults who have a relationship with the child. The CARE-Index has proven effective not only for the screening of risky conditions in parents, but also as a video-feedback technique to evaluate the results of treatment [88].
- Lausanne Trilogue Play (LTP) video feedback is a tool with diagnostic and therapeutic implications [89,90]. It is based on the assumption that family dynamics cannot simply rely on the dyadic components, since they are better understood when considering the family as a unit. The LTP video feedback consists of a sort of “family game” and it allows the observation of family interactions in the triadic relationship (father-mother-child). The goal of the triadic game is to investigate the capacity of affective regulation, sharing, and empathic responsiveness. The session is divided in four phases as follows: First, the mother and the child play together, while the father assumes a peripheral position; in the second phase the father and the child play together and the mother stands apart; then, the three play together; lastly, the child is in a peripheral position, while the parents talk to each other. The setting requires family members to be placed as if they were at the top of an equilateral triangle with both parents maintaining an “equal” position with respect to the child. The entire session is then videotaped and, subsequently, codified through four levels graded on a three-point scale, i.e., participation (“are they all participating?”), organization (“does everyone respect his role?”), focal attention (“are they all attentive to the activity in progress?”), and emotional contact (“is there an emotional sharing?”). Parents can review the video and discuss the session which can increase the awareness of their positive and negative styles of interaction. Thus, video feedback provides a double perspective on family functioning, the experience of real-time interaction and the experience at a distance of time.

### 9.2. Mindfulness-Based Interventions

More recently, several studies have evidenced a reduction of depressive symptoms after mindfulness-based programs [97]; according to these findings, PPND also benefits from such interventions [98]. The researchers proposed a “conscious parenting” model based on mindfulness, and focused on several dimensions concerning the parent-child relationship which included: present moment attentiveness, non-judgmental acceptance, non-reactivity, emotional awareness, self-regulation of the parental relationship, and compassion for oneself and for one’s child [99]. It was shown that parents who learn mindfulness practice develop an attitude towards a high-quality relationship with their children [99,100]. According to the stress and coping theory, people have different experiences when faced with stressful events, such as parenthood [101]. Some people consider it dangerous or harmful, whereas others view it as a challenge. Considering parenthood as a challenge rather than a threat offers the couple the possibility to experience positive feelings and to develop more adaptive coping strategies. Some authors applied a “Mindfulness-Based Childbirth and Parenting” (MBCP) program [102], an adaptation of the “Mindfulness-Based Stress Reduction” program [103] whose purpose was to improve the impact of the stress-induced changes occurring during pregnancy, childbirth, and parenting. The MBCP protocol is composed of ten weekly meetings lasting about three hours. Furthermore, participants are encouraged to engage in meditation at home for 30 min a day. Mindfulness learning is implemented with information about the psychological processes of pregnancy, birth, breastfeeding, postpartum, and the needs of the newborn. Participants also learn to understand how “non-reactivity”, concentration, and calm can be useful to allow unpleasant sensation to emerge and disappear naturally.

## 10. Conclusions

The findings from this review on PPND should be interpreted within the context of a number of limitations. First, PPND is not an officially codified psychiatric disorder, and it has no basis in any diagnostic manual. The lack of an explicit set of diagnostic criteria has led to certain heterogeneity in PPND construct and definition. Second, there are limitations associated with the varying assessment methods; psychodiagnostic instruments and cut-off scores used among studies could have contributed to the observed variability in prevalence rates. Therefore, the variability could be due to the use of different research methods and to the lack of standardized measurements or cut-off scores, along with the almost exclusive use of self-report scales that do not acknowledge the possible differences in how men and women express mood symptoms. All these factors contribute to a lack of rigor and bias in the assessment of depressive symptoms, and possibly result in underestimation of fathers’ depression levels. Third, the time of assessment of PPND in the examined studies may not perfectly capture the peculiar clinical features of the disorder; the majority of studies have provided single time-point observations or short-term follow-ups that significantly limit the understanding of the long-term effects of PPND in the affected individuals. Finally, this review was limited to peer-reviewed articles. Inclusion of the so-called “gray literature” and otherwise unpublished dissertation and theses would have added further knowledge on this topic.

Despite the limitations, this study provides an overview of PPND, summarizing current and emerging evidence for heterogeneity in prevalence rates, symptom profile, risk factors, and symptom trajectories. Substantial attention should be paid to those subjects who constitute a group particularly at risk of PPND, such as those fathers pertaining to “non-traditional settings”, such as stay-at-home dads, single fathers, or nonbiological fathers. Longitudinal studies should better define PPND outcomes and long-term effects on the fathers’ and family’s functioning and relationships. International and intercultural studies would highlight possible differences or similarities in PPND symptoms and clinical presentations in different countries, along with differences in stigmatization and cultural beliefs. Finally, neurobiological and brain-imaging studies are needed to achieve a wider knowledge of the involved brain circuits and neurohormonal systems. The early recognition of depressive and negative emotional symptoms in fathers requires a specific knowledge of this issue, a sensitive and informed clinical approach, more accurate and specific diagnostic instruments, and interventional programs addressed at treating paternal depression, a clinical disorder with significant negative repercussions on individuals, couples, families, and infant development and health.

## Figures and Tables

**Table 1:** Etiopathogenetic hypotheses and risk factors for paternal perinatal depression.

Psychodynamic Hypotheses	Psychosocial Risk Factors	Biological Risk Factors
- Reactivation of pre-oedipal or oedipal conflicts - Activation of men’s defense to cope with the anxiety caused by the birth of a child - Regressive attitudes of dependence and jealousy due to rivalry towards one’s child - Incestuous unconscious fantasies related to a conflict with their own mother - Identification with one’s father - Substantial dependence on their partners - Conflicts linked to latent homosexuality - Defense against the reactivation of memories of a distressing childhood - Envious unconscious responses to the partner’s generative role expressed through competitive and devaluating attitudes - Fear of growing old or dying - Fear of losing the partner’s attention (the child is perceived as a rival)	- The manifestation of depressive symptomatology in the mother - Presence of couple conflicts and marital dissatisfaction - Being unemployed or having work-family conflicts - Perceived stress level - Psychological characteristics (depressive traits, neuroticism, low level of extraversion, immature defensive styles, low self-esteem) - Childhood history (low educational level, history of physical or sexual abuse) - Poor social support - Insufficient information about childbirth and pregnancy - Young or advanced age - Unfulfilled expectations related to the birth (e.g., the desire for a child of different sex) - Perception of poor parenting skills - Previous abortions or the death of a child - Unexpected pregnancy	- Low testosterone levels - Reduction of estrogen levels - Lower cortisol levels - Lower levels of vasopressin - Reduction of prolactin
